# 

**Published:** 1898-02-05

**Authors:** 


**Notices of Births, Deaths, and Marriages** are inserted in this column at a charge of 2s. 6d. for an announcement not exceeding four lines.

### NOTICE TO CORRESPONDENTS.

All MS., Letters, Books for Review, and other matters intended for the Editor should be addressed The Editor, "The Hospital," The Hospital Building, 28 & 29, Southampton Street, Strand, W.C.

The Editor cannot undertake to return rejected MS., even when accompanied by stamped directed envelope.

**Notice to Advertisers and Subscribers.**

All Advertisements, Orders for Copies of The Hospital, and Business Communications should be addressed to **THE MANAGER, (Not to The Editor),** The Hospital, The Hospital Building, 28 & 29, Southampton Street, Strand, London, W.C.

The Cost of Subscription, post free, is as follows:—Per week, 2½d. per quarter, 2s. 8d.; per half-year, 5s. 3d.; per annum, 10s. 6d. Foreign:—Per annum, 15s. 2d.; payable, in all cases, in advance.

**NOTICE.—Vol. XXII. of "The Hospital" (April, 1897, to September, 1897), in handsome cloth binding, is now on sale at the Office of this Journal, price 7s. 6d. Cases for binding the Numbers contained in this Volume. 1s. 6d. Index to Vol. XXII., 2½d., post free. The Monthly Part for February is now ready. Price 6d., by post 8d.**

### NOTICE.

We have delayed the publication of The Hospital this week for one day, in order to include a report of the first annual meeting of the Council of the Prince of Wales's Hospital Fund for London. So much interest is felt in this important Fund that we feel sure our readers will appreciate our action in securing to them a full account of this work and its progress during the past year, which was reported for the first time at Thursday's meeting.

### Scraps and Gleanings.

A cottage hospital for Carshalton has been proposed.

Cork Hospital Saturday collection in 1897 amounted to £431.

The new Eye, Ear, and Throat Hospital at Cork has recently been opened.

A new hospital for women is now in process of being built on the Dundee Infirmary grounds.

The number of patients treated at the Cotswold Convalescent Home was larger in 1897 than in 1896.

A new operating theatre was opened towards the latter end of last year at the Dunedin Hospital, New Zealand.

The Hull Orthopædic Hospital in the year 1896-7 treated 121 in and 3,667 out patients, an increase on former years.

During last year 76 patients were treated at the Holmesdale Cottage Hospital. About £420 was received during the year, and £454 expended.

Sixty patients were treated at the Brixham Cottage Hospital in the year 1896-7. The district cases numbered 593, and included 158 maternity cases.

Princess Christian has consented to become a patron of the Children's Cottage Hospital at Cold Ash. Sixty children were treated in 1896-7.

The total number of attendances on out-patients at the Manchester Ear Hospital in 1896-7 was 9,507, the number of new patients being 1,990. In-patients numbered 128.

The question of providing a new workhouse infirmary at Bath has been referred back by the Guardians to the General Purposes Committee for further consideration.

Compared with 1894 the total collections of the Birmingham Hospital Sunday Fund in 1897 show an increase of over £600, the respective figures being £4,539 and £5,178.

In-patients at the Tynemouth Victoria Jubilee Infirmary numbered 167 in 1896-7, and out-patients numbered 141. There was a decrease in the number of patients treated of 57.

The committee appointed to collect funds for the Cottage Hospital at Castle Douglas, Galloway, have reached the practical stage of asking for plans and selecting a site for the hospital.

Of the £571 collected by the Royal Lancaster Infirmary Workpeople's Committee, £460 resulted from weekly contributions from workpeople, and £54 from Hospital Saturday collections.

As a result of Sir Squire Bancroft's reading of the "Christmas Carol" in aid of the Jewish Home and Hospital for Incurables, a sum of about £250 was handed over to the institution.

During the year 1896-7 146 in-patients and 1,300 out-patients were treated at the Dublin Orthopædic Hospital. A house was secured during the year at Templeogue to serve as a convalescent home.

For three successive years the committee of the Norwich Hospital Saturday and Sunday Fund have had to announce that the collection for the year has been the largest on record. In 1897 the collection reached £1,484, compared with £1,303 in 1896.

The cost per in-patient at the Victoria Infirmary, Glasgow, was £4 9s. 3d. in 1896-7, as compared with £4 9s. in 1895-6. The report of the infirmary mentions the purchase of a house, situated at the northern end of Largs, for the purposes of a convalescent home.

Fourteen hospitals and dispensaries were open in the Presidency Town of Madras at the end of 1896, during which year 17,605 in and 186,249 out-patients were treated. One thousand three hundred and nine beds were available for patients, of which a daily average number of 975 were occupied.

The ordinary income of the Coventry and Warwickshire Hospital amounted in 1896-7 to £3,349, against an ordinary expenditure of £3,756. Six hundred and twenty-four in-patients were treated, against 616 in the previous 12 months, and 5,878 out-patients were under treatment, compared with 5,693 in 1895 6.

The committee appointed to deal with the collections on Hospital Saturday and Sunday for 1897 in Southampton state in their report that the total amount collected had been £1,175. From that sum was to be deducted £57 incidental expenses, leaving a balance of £1,118 to be divided, as against £1,119 last year.

Now that it has been decided, if permission is granted by Parliament, to give a free site on Corporation property for the Royal Victoria Hospital at Belfast, the Mater Infirmiorum Hospital has applied to the Corporation to grant them the capitalised value of the rent they have to pay for their site, which is also on Corporation ground, as the equivalent of a free site.

In the report of the Birmingham Hospital Sunday Fund for 1897 it is mentioned that in the case of one church a collection for the movement was made, and, although the amount was small, instead of the money being forwarded to the treasurer in the ordinary way, it was utilised in the purchase of dispensary or hospital tickets, thus, of course, not in any way benefiting the Hospital Fund.

